# Development of an immunodeficient pig model allowing long-term accommodation of artificial human vascular tubes

**DOI:** 10.1038/s41467-019-10107-1

**Published:** 2019-05-21

**Authors:** Manabu Itoh, Yosuke Mukae, Takahiro Kitsuka, Kenichi Arai, Anna Nakamura, Kazuyoshi Uchihashi, Shuji Toda, Kumika Matsubayashi, Jun-ichi Oyama, Koichi Node, Daisuke Kami, Satoshi Gojo, Shigeki Morita, Takahiro Nishida, Koichi Nakayama, Eiji Kobayashi

**Affiliations:** 1 Department of Thoracic and Cardiovascular Surgery, Faculty of Medicine, Saga University, Saga, Japan; 2 Department of Regenerative Medicine and Biomedical Engineering, Faculty of Medicine, Saga University, Saga, Japan; 3 Department of Surgical Pathology, National Hospital Organization Saga Hospital, Saga, Japan; 4 Department of Pathology & Microbiology, Faculty of Medicine, Saga University, Saga, Japan; 5 Cyfuse Biomedical K. K., Tokyo, Japan; 6 Department of Cardiovascular Medicine, Faculty of Medicine, Saga University, Saga, Japan; 7 Department of Regenerative Medicine, Kyoto Prefectural University of Medicine, Kyoto, Japan; 8 Department of Cardiovascular Surgery, National Hospital Organization Kyushu Medical Center, Fukuoka, Japan; 9 Department of Organ Fabrication, Keio University School of Medicine, Tokyo, Japan

**Keywords:** Tissue engineering, Transplant immunology, Cardiovascular biology

## Abstract

Before they are used in the clinical setting, the effectiveness of artificially produced human-derived tissue-engineered medical products should be verified in an immunodeficient animal model, such as severe combined immunodeficient mice. However, small animal models are not sufficient to evaluate large-sized products for human use. Thus, an immunodeficient large animal model is necessary in order to properly evaluate the clinical efficacy of human-derived tissue-engineered products, such as artificial grafts. Here we report the development of an immunodeficient pig model, the operational immunodeficient pig (OIDP), by surgically removing the thymus and spleen, and creating a controlled immunosuppressive protocol using a combination of drugs commonly used in the clinical setting. We find that this model allows the long-term accommodation of artificial human vascular grafts. The development of the OIDP is an essential step towards a comprehensive and clinically relevant evaluation of human cell regeneration strategies at the preclinical stage.

## Abstract (web summary)

The development of tissue-engineered vascular grafts heavily relies on the availability of large animal models that allow long-term assessment of graft patency. Here Itoh et al. propose a novel model of immunodeficient pigs that allows long-term accommodation of human cell-derived three-dimensional bioprinted vascular tubes.

## Introduction

As various powerful immunosuppressive agents were developed in the 1980s, the results of organ transplantation in humans have dramatically improved, resulting in an increased need for organs for transplantation. On the other hand, under the current situation of a worldwide donor organ shortage, the production of human organs has become an important objective of regenerative medicine^1–3^. In recent years, there have been remarkable developments in stem cell technology and tissue engineering technology such as induced pluripotent stem cells. Meanwhile, three-dimensional (3D) bioprinting technology has also been developed, which allows for the construction of any 3D structure using cells alone, without scaffold materials^4,5^, and we successfully developed the human original 3D bioprinted tube (HOBPT)^6^ using our original 3D bioprinting system. Although this system has enabled the production of organs that can be implanted into small animals, such as vascular grafts^6^, substitute nerves^7^, liver^8^, and myocardium^9^ using human-derived cells, the development of large animal models to be used for preclinical studies to evaluate the efficacy and safety of the implantation of human organs produced using this technology is currently underway.

Mini-pigs are superior in terms of their reproductive capacity as multiple pregnancies are possible. Furthermore, they are well-tempered and easy to handle. Moreover, they are similar to humans in terms of their anatomy, physiology, immune system, and lifespan. Given these advantages, the mini-pig immunodeficiency model is expected to be a sufficient large animal model enabling evaluation after the transplantation of human stem cells^10–13^. Combination immunosuppressive therapy, which was established based on the use of immunosuppressive therapy in clinical practice^14,15^, has also been administered to mature mini-pigs for preclinical studies after human-derived cell transplantation. However, it is generally impossible to control immunosuppression over several months owing to a strong immune response against human cells or tissues. A severe combined immunodeficiency (SCID) pig model was developed using genetic modification technology; however, unlike SCID mice, long-term feeding was found to be difficult even under sterile conditions, which represented a major problem^16–18^. Furthermore, although T-cell immunodeficiency can be induced by neonatal total thymectomy in rodents, we have already proved that neonatal total thymectomy alone was not sufficient for inducing complete immunodeficiency in pigs^19^.

In the present study, we evaluate the long-term results and immunosuppressive effects after xenografting the HOBPT as a neck arteriovenous shunt graft in our original operational immunodeficient pig (OIDP) model, created by removing the major immune organs and the controlled administration of combination immunosuppressive therapy under careful monitoring. We then compare the results to those in a conventional immunosuppressive pig (CISP) model in which immunosuppression is controlled by combination immunosuppressive therapy alone. Our OIDP model demonstrates the long-term accommodation of the HOBPT, whereas CISP model suffers from severe rejection. The development of OIDPs without the rejection of human cell-derived organs symbolizes an essential step toward the comprehensive evaluation of preclinical cell regeneration strategies and contributes to the development of regenerative medicine using human organs.

## Results

### Development of the OIDP model

**Fig. 1:**
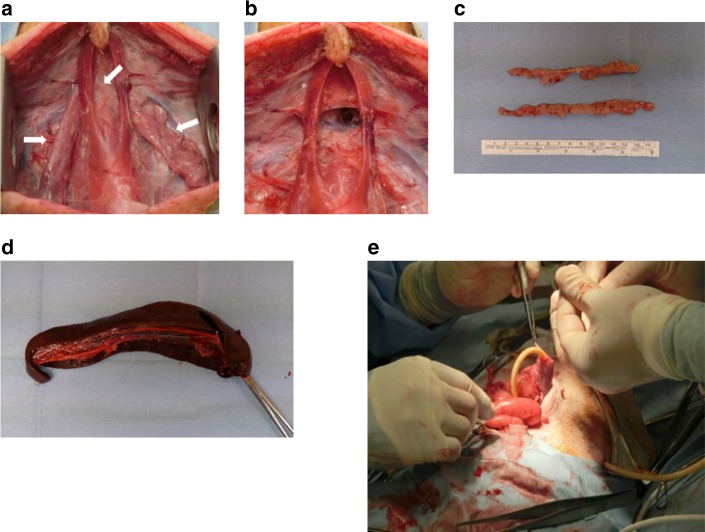
Thymectomy, splenectomy, and gastrostomy of a mini-pig. The image of the ventral cervical region of the neck before thymectomy. The white arrow indicates the lobes of the thymus **a**. An image of the ventral cervical region of the neck after thymectomy **b**. An image of the resected thymus **c**. An image of the resected spleen **d**. An image of gastrostomy performed under laparotomy **e**

Six Göttingen mini-pigs (male; age, 6–7 months; weight ≥ 15 kg; Ina MP Production Center, Oriental Yeast Co., Ltd.) were anesthetized with 0.5–1.5% isoflurane inhalation anesthesia. A midline incision was made on the ventral region of the neck, and three lobes of the thymus (the left and right cervical lobes and the thoracic lobe) were excised. Subsequently, laparotomy was performed by making an incision in the middle of the abdomen to remove the spleen. At the same time, the upper portion of the stomach was exposed with the insertion of a catheter (16 F, JMS hydrophilic Foley catheter, JMS), after which the stomach and catheter insertion site was ligated and fixed at the upper portion of the stomach to prevent contact with the ground to avoid abscess formation around the gastrostomy. One end of the catheter was exposed to the median line of the back through the skin on the right side and sutured and fixed to the skin (Fig. 1). Tacrolimus hydrate (0.5 mg/kg/day), mycophenolate mofetil (60 mg/kg/day), and prednisolone (20 mg/body/day) were administered through the gastric tube from the day after removal of the major immune organs. An HOBPT was transplanted at 1 week after the removal of the major immune organs and the blood tacrolimus and mycophenolic acid concentrations were measured (measurement location: SRL, Inc.). The dose was adjusted so that the trough levels of tacrolimus and mycophenolic acid in the blood were 10–20 ng/mL and 2–6 μg/mL, respectively. Ampicillin sodium (1 g/5 mL/body) and buprenorphine hydrochloride (0.01 mg/kg; liquid quantity 0.05 mL/kg) were intramuscularly administered for 3 days from the date of transplantation. The blood concentrations of tacrolimus and mycophenolic acid tended to fluctuate according to dosage. There were no deaths or serious complications, including infection, liver, or renal dysfunction during the months after transplantation (Supplementary Fig. 1, Supplementary Data 1–6).

The CISP group (*n* = 6) received the same drug therapy as the OISP group from 6 days before transplantation, without organ removal. After the removal of the HOBPT, pentobarbital sodium (5 mg/kg; liquid quantity, 0.1 mL/kg) was administered, and the pigs were deeply anesthetized and then killed by exsanguination via the abdominal aorta.

Both the OISPs and CISPs were maintained under the same environment in Good Laboratory Practice (GLP) institute to apply as a preclinical trial. They were housed individually in stainless steel cages (*W*: 1190 × *D*: 590 × *H*: 720 mm) placed in an animal room on a 12 h light and dark cycle (lighting: 06:00–18:00) with a temperature range of 22–24 °C and a relative humidity range of 43–84%. The environment is maintained with a high-performance clean system that evokes once every 10 min, and it has a barrier system. According to the protocol, the animals were given 350 g of solid feed (MP; Oriental Yeast Co., Ltd., Tokyo) per day. The immunosuppressive agents were administered from a catheter via gastrostomy in OISPs, on the other hand, they were administered with its feed in CISPs.

### Scaffold-free HOBPTs

**Fig. 2:**
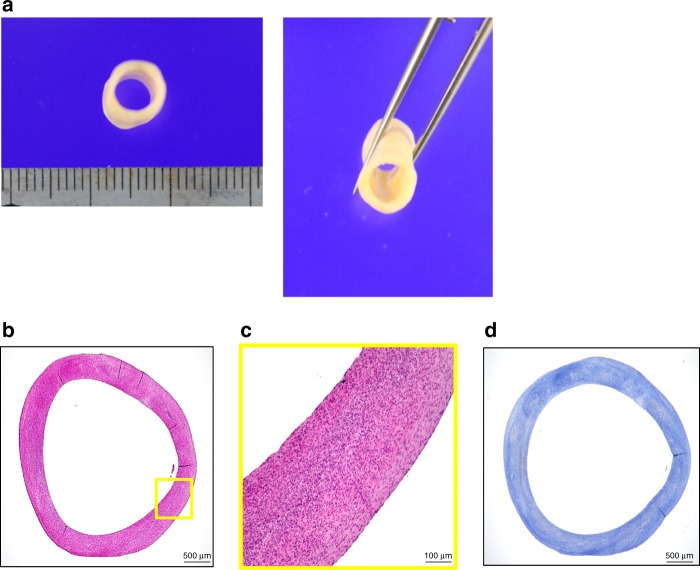
Macroscopic photographs and micrographs of the HOBPT. The HOBPT had an inner diameter of 5 mm and a soft, elastic texture **a**. Microscopically, the HOBPT was composed of human fibroblasts and collagenous extracellular matrix **b**, **c**, stained blue by Masson–trichrome staining **d**. No endothelial cells were seen on the luminal surface of the HOBPT **c**. Scale bar: 500 µm in **b**, **d**, 100 µm in **c**. HOBPT: human original 3D bioprinted tube

We created scaffold-free HOBPTs with an inner diameter of 5 mm (Fig. 2a) using a Bio-3D printer that we originally developed. Histologically, the HOBPTs were composed of human fibroblasts and collagenous extracellular matrix without an endothelial cell lining (Fig. 2b–d).

### Patency and morphological changes in the HOBPTs

HOBPTs were xenotransplanted into the OIDP group (*n* = 6) and the CISP group (*n* = 6) as a substitute blood vessel of the neck arteriovenous shunt.

In the OIDP group, the HOBPT was removed at 2 weeks (*n* = 2), 1 month (*n* = 2), and 3 months (*n* = 2) after transplantation. We performed an ultrasound examination using a Hi Vision Preirus ultrasonography system (Hitachi, Japan) every 2 weeks after transplantation and at the time of removal; patency was evaluated using angiography. The ultrasound examination revealed that the inner graft diameter was dilated from 5.0 to 8.5 mm and extended in a longitudinal direction from 10 to 12 mm at 1 month after transplantation (Supplementary Fig. 2a). The wall thickness of the HOBPT gradually increased from 1–3 months after transplantation (Supplementary Fig. 2b). No obstruction occurred during the observation period.

In contrast, in the CISP group (*n* = 6), we confirmed that all HOBPTs were occluded within 2 weeks, and these occluded vessels were removed. The wall thickness of the graft was increased at 2 weeks after transplantation (Supplementary Fig. 2b). No findings of rupture or aneurysm were observed in either group.

### Rejection after HOBPT transplantation in CISPs

**Fig. 3:**
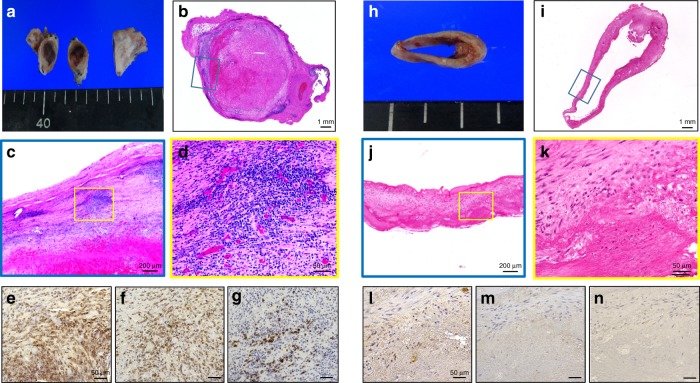
A histological analysis of the patent or rejected HOBPTs at 2 weeks after transplantation. The lumen of the HOBPT in the CISP group was completely obstructed by thrombus **a**, **b**, but was patent in the OIDPs **h**, **i**. The lymphocyte infiltration in the HOBPT of the OIDP group **j**, **k** was drastically inhibited in comparison with the CISP group **c**, **d**. Immunohistochemically, the numbers of CD45 + common lymphocytes **e**, **l**, CD20 + B lymphocytes **f**, **m** and CD3 + T lymphocytes **g**, **n** in the OIDP group **l–n** were significantly decreased in comparison with the CISP group **e–g**. Scale bar: 1 mm in **b**, **i**, 200 µm in **c**, **j**, 50 µm in **d–g**, **k–n**. HOBPT: human original 3D bioprinted tube, CISP: conventional immunosuppressive pig, OIDP: operational immunodeficient pig

**Fig. 4:**
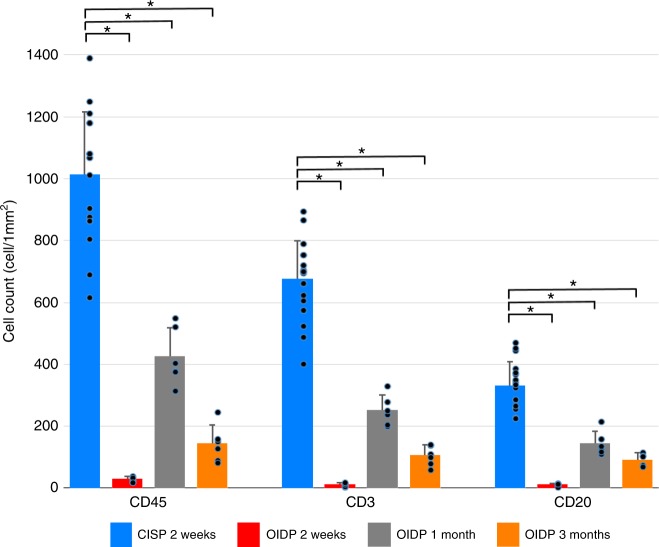
The evaluation of lymphocyte infiltration according to the cell count. The numbers of CD45 + common lymphocytes, CD20 + B lymphocytes, and CD3 + T lymphocytes in the OIDP group were all significantly decreased in comparison with the CISP group at 2 weeks to 3 months after transplantation. (CISP group: *n* = 6, each OIDP group: *n* = 2) The s.d. is reported as error bar, with paired symbols indicating differences with *P* < 0.05 (vs CISP 2 weeks) using Student’s *t* test. CISP: conventional immunosuppressive pig. OIDP: operational immunodeficient pig

At 2 weeks after transplantation, the lumen of the HOBPT was completely obstructed by thrombus in the CISPs (Fig. 3a, b), whereas it was patent in the OIDPs (Fig. 3h, i). Histologically, numerous mononuclear lymphoid cells had infiltrated the HOBPT in CISPs (Fig. 3c, d), whereas the infiltration in the OIDP group was drastically inhibited (Fig. 3j, k). Immunohistochemically, the numbers of CD45 + common lymphocytes, CD3 + T lymphocytes and CD20 + B lymphocytes in the OIDP group were significantly decreased in comparison to the CISP group at 2 weeks to 3 months after transplantation (Figs. 3e–g, l–n, 4).

### The histological analysis of the HOBPT in OIDPs

**Fig. 5:**
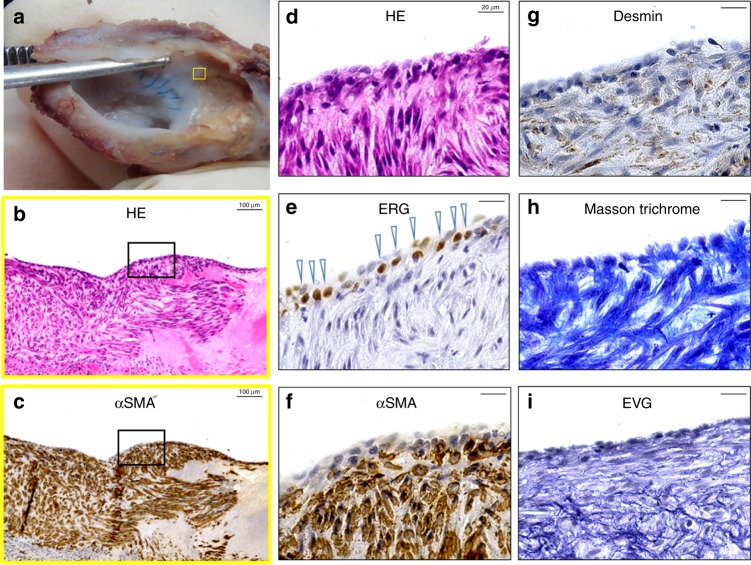
The histological analysis of the HOBPT at 1 month after transplantation in OIDPs. The luminal surface of the HOBPT was whitish, smooth and contiguous from the surface of the recipient’s common carotid artery without thrombus **a**. Microscopically, the luminal surface was lined with a monolayer of flat cells **b**, **d** expressing ERG (an endothelial cell marker) **e**. Host-derived spindle-shaped cells migrated into the HOBPT wall from the upper, lower, and lateral margins **b**, and the cells expressed the smooth muscle cell markers alpha-SMA **c**, **f** and desmin **g**. Elastica van Gieson staining showed “neomedia” containing elastic fibers beneath the luminal surface **i** in the collagenous extracellular matrix, which was stained blue by Masson–trichrome staining **h**. Scale bar: 100 µm in **b**, **c**, 20 µm in **d–i**. HOBPT: human original 3D bioprinted tube, OIDP: operational immunodeficient pig

**Fig. 6:**
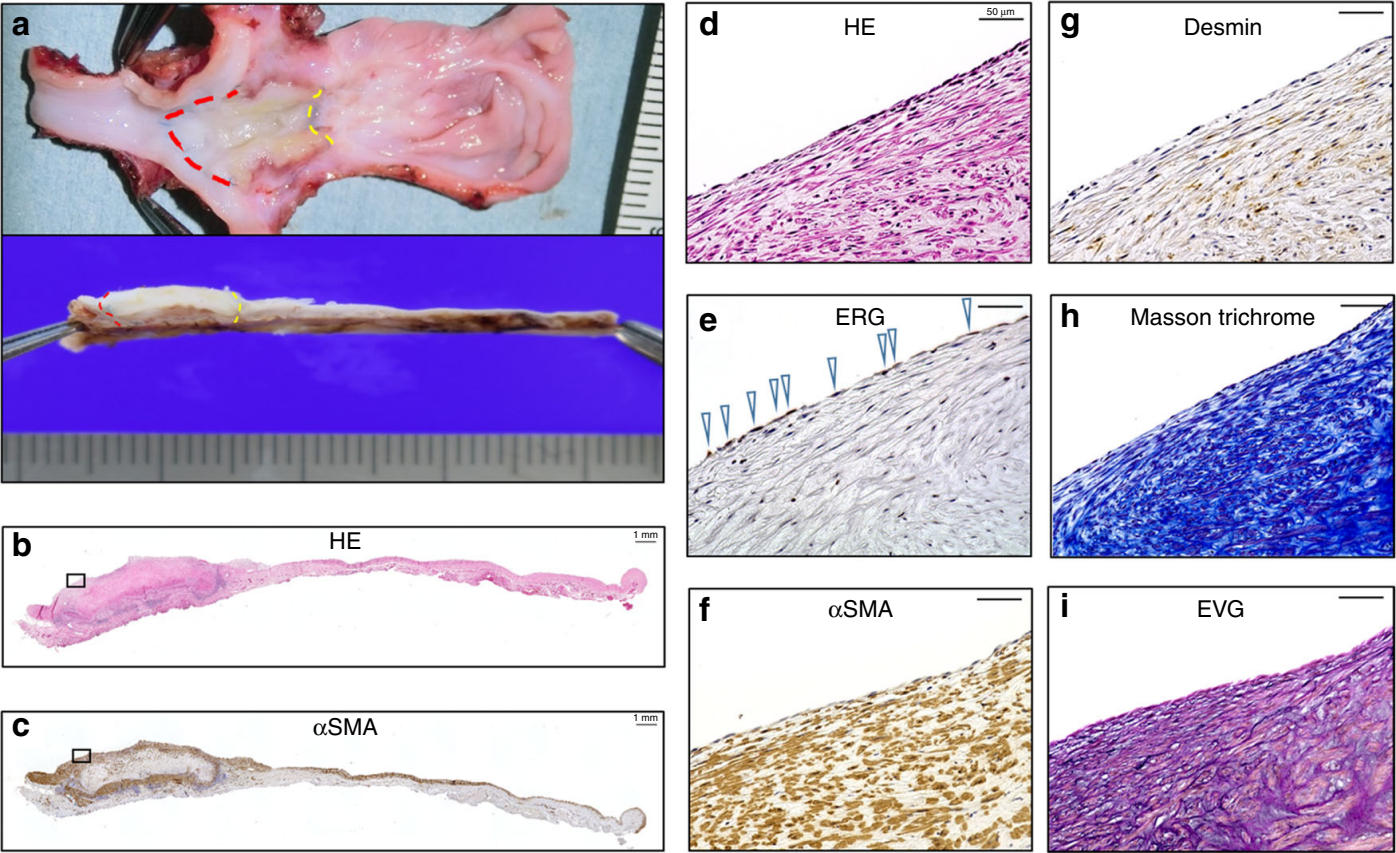
The histological analysis of the HOBPT at 3 months after transplantation in OIDPs. The graft was opened longitudinally with the proximal artery and distal vein. The red dotted line indicates the junction of the artery and graft, and the yellow dotted line indicates that of the graft and vein. The luminal surface of the HOBPT was whitish, smooth, and contiguous from the surface of the recipient’s common carotid artery and vein without thrombus **a**. Microscopically, the luminal surface was lined with an ERG-positive monolayer of endothelial cells **b**, **d**, **e**. Host-derived alpha-SMA and desmin-positive smooth muscle cells migrated into the HOBPT wall from the upper, lower and lateral margins **b**, **c**, **f**, **g**. Elastica van Gieson staining showed “neomedia” beneath the luminal surface **i** in the collagenous extracellular matrix and stained blue by Masson–trichrome staining **h**. Scale bar: 1 mm in **b**, **c**, 50 µm in **d**–**i**. HOBPT: human original 3D bioprinted tube, OIDP: operational immunodeficient pig

Macroscopically, the luminal surface of the HOBPT was whitish and smooth without thrombus and was contiguous from the surface of the recipient’s common carotid artery at 1 and 3 months after transplantation (Figs. 5a, 6a). Microscopically, the luminal surface was lined by a monolayer of flat cells expressing endothelial cell markers ERG (Figs. 5e, 6e). In addition to endothelial cells, spindle cells migrated into the HOBPT wall from the upper, lower and lateral margins, and the cells expressed smooth muscle cell markers alpha-SMA (Figs. 5c, f, 6c, f), and desmin (Figs. 5g, 6g) indicating a host-derived smooth muscle cell origin. Furthermore, thin wavy fibers that were black on Elastica van Gieson staining, suggesting neomedia formation, were generated beneath the lumen after transplantation (Figs. 5i, 6i), although the extracellular matrix was mainly composed of collagen fibers stained blue by Masson–trichrome staining (Figs. 5h, 6h), similar to the HOBPT before transplantation (Fig. 2d). Endothelial cells and smooth muscle cells were observed to have migrated more extensively at 3 months after transplantation than at 1 month after transplantation (Figs. 5a–c, 6a–c). Scanning and transmission electron microscopy revealed an endothelial-like cell lining on the luminal surface of the HOBPT (Supplementary Fig. 3a, b).

### Peripheral blood leukocytes and serum immunoglobulin

The respective numbers of neutrophils, lymphocytes, and monocytes were 10,260 ± 1796 cell/µl, 5125 ± 219 cell/µl, and 1025 ± 7 cell/µl in the CISP group and 265 ± 290 cell/µl, 3520 ± 112 cell/µl, and 120 ± 85 cell/µl in the OIDP group at 2 weeks after implantation (average ± range, *n* = 2) (Supplementary Fig. 4a). The respective serum IgG and IgM concentrations were 4970 ± 2810 µg/ml, 1637 ± 123 µg/ml in the CISP group and 3467 ± 958 µg/ml, 1053 ± 70 µg/ml in the OISP group at 2 weeks after implantation (average ± range, *n* = 2) (Supplementary Fig. 4b). Both the number of inflammatory cells and the serum levels of immunoglobulin were suppressed in the OIDP group compared with the CISP group.

## Discussion

In this present study, we clearly showed that we could successfully induce an immunodeficient state in mature mini-pigs in which long-term feeding was enabled by the surgical removal of the major immune organs, including the thymus and spleen, and by adjusting the immunosuppressive agents based on the physical condition of the pigs (OIDP group; Fig. 1, Supplementary Fig. 1). Using our OIDP model, we confirmed that all HOBPTs were patent in the OIDP group. In contrast, all HOBPTs were occluded within 2 weeks in the CISP group (used as a control group) owing to acute resection.

Histopathological examinations during the acute phase, at 2 weeks after transplantation revealed that inflammatory cell infiltration (including CD3 + lymphocytes) was markedly suppressed in the OIDP group (Fig. 4). The patency was maintained for 3 months in two of six pigs in the OIDP group. There were fewer lymphocytes infiltrating into the HOBPT at 3 months after transplantation than at 1 month post surgery. Given that the histology of HOBPT at 3 months after transplantation showed that host-derived smooth muscle cells had extensively migrated into the HOBPT wall (Figs. 5a–c, 6a–c), the inflammatory phase seems to have shifted to the remodeling or fibrosing phase.

It should be noted that this is the first report in which tissue-engineered grafts derived from human cells remained patent for > 1 month after xenotransplantation in a large animal model. On the other hand, occlusion occurred at ~ 2 weeks in all six pigs of the CISP group (Fig. 3a, b), and remarkable inflammatory cell infiltration was observed in the tissues (Fig. 3c, d). These results demonstrate the severity of acute rejection, as reported in previous studies of xenotransplantation of tissue-engineered grafts derived from human cells under triple drug immunosuppression therapy^20^. Our data of peripheral blood leukocytes and serum immunoglobulin after HOBPT implantation indicate the histological differences in the inflammatory reaction (Supplementary Fig. 4a, b) between the two groups and suggest that controlling systemic circulating inflammatory cells and antibodies is important for ensuring graft patency^21,22^.

Examining the morphological changes in the graft after transplantation is also important when evaluating ongoing graft rejection. The graft dilates owing to the pressure and volume load experienced under a physiological environment, so we evaluated the morphology of the HOBPTs by an ultrasonic examination (Supplementary Fig. 2). We assumed that the acute wall thickening of the HOBPTs in CISP was owing to acute rejection, whereas the gradual wall thickening in OIDP was owing to the pressure and volume load because it was similar to the physiological response of native vessels in the arteriovenous shunt environment^23^.

Immunodeficient mice can be created by genetic recombination technology and several studies have been conducted, not only for drug discovery but also for preclinical studies of new drugs, basic research differentiation and the functional analysis of human cells^24,25^. However, the findings from studies of transplantation of clinical relevant organs prepared from human cells are not necessarily applicable to humans, owing to differences in size, lifespan, the number of cells, and the duration for preparation, along with the observation period after transplantation. In this context, an immunodeficient large animal model is required to evaluate medical bio-material products for human use. However, among SCID pigs, 45% of fetuses are stillborn, with most of the survivors being difficult to manage, and dying on the 70th day of life, even under sterile conditions^17^. In addition, the costs and time associated with producing SCID pigs are enormous. To overcome these limitations, it was necessary to develop another type of immunodeficient pig model. It has been reported that the immunosuppressive effect thymectomy alone is insufficient for inducing immunosuppression in young piglets^10^. Thus, we removed the thymus and the spleen, which are the major immunological organs, and simultaneously carried out gastrostomy in order to enable the administration of the immunosuppressive agents, regardless of dietary intake. It should be emphasized that we made the gastrostomy in a higher position of the stomach to avoid abscess formation around the gastrostomy because we had already confirmed that gastrostomy at a lower position of the abdomen (nearer to the ground) was easily and severely infected in quadruped pigs in an immunosuppressive state. With the use of our controlled immunosuppressive therapy via a higher positioned gastrostomy, we could sufficiently control immunization as well as infection by the administration of general antibiotics in the acute phase after transplantation, without breeding in special sterile conditions such as a specific pathogen-free (SPF) environment. The cost of maintaining a SPF environment for a long time is substantial. Therefore, the fact that OIDP animals do not require SPF management is a great advantage in terms of their management, as well as the cost and time associated with the manufacture of SCID pigs. Another major advantage of the OIDP model is the fact that this model can be universally adapted to “maturation” pigs by adjusting the amount of immunosuppressant according to the type of pig. Because the age of the pig is also one important factor influencing the response for the verification of human-derived cell products^26^.

This study clearly demonstrated the usefulness of OIDP as well as the excellent outcome of our 3D bioprinting technology in the development of small-diameter artificial blood vessels. Existing small-diameter ( < 6 mm) artificial blood vessels made of foreign substances such as PTFE (polytetrafluoroethylene) have many problems that remain to be solved, including—but not limited to—anti-thrombogenicity, anti-infectivity, and biocompatibility^27,28^. In recent years, there have been reports on the development of small-diameter artificial blood vessels using tissue engineering techniques with different approaches, including artificial blood vessels using biodegradable polymers as scaffolds or artificial blood vessels made of decellularized vascular tissue^29–31^. On the other hand, 3D bioprinting technology developed by Nakayama et al. has many advantages because small-diameter artificial blood vessels of various sizes can be prepared using autologous cells alone, without any scaffolding material, which is a foreign substance. As the ultimate goal of our study is the clinical application of this vascular structure, it is necessary to use all materials, including the medium, according to the regulatory guidelines of biological products for human use. Porcine cell-derived constructs made with these media were unable to attain the desired strength because the types of products that meet such criteria are not available on the market. Thus, the development of our OIDP model is crucial for the comprehensive evaluation of human original products at the preclinical stage.

Although HOBPTs were originally only produced from human skin-derived fibroblasts, a post-transplantation histopathological examination in the OIDP group revealed replacement with several mini-pig derived cells in the grafts, including vascular endothelial cells (Figs. 5e, 6e), vascular smooth muscle cells (Figs. 5c, f, 6c, f), and extracellular matrix, including elastic fibers (Figs. 5i, 6i). These results strongly indicate the self-organizing phenomenon as a living blood vessel derived from a recipient (mini-pig) after transplantation and that long-term accommodation may be achieved through this form of “adjustable” inflammation-induced regeneration. These self-organizing phenomena induced by the interaction and migration of host-derived cells constituting the original vital blood vessels might continuously improve the physiological function of HOBPTs over the long-term because the physiological function as well as anti-thrombogenicity of the original vital blood vessels may be maintained and continue to accommodate the HOBPT to the original blood vessels. If this HOBPT becomes clinically available, its application can be expected not only as artificial blood vessels for dialysis access, which are problematic owing to puncture site infection and thrombus trouble, but also as an alternative small-diameter artificial blood vessel substitute for autologous vein grafts with potential applications in coronary artery bypass surgery or bypass surgery at the distal lower extremities.

By surgically removing the major immune organs and implementing controlled therapeutic strategies with combination immunosuppression, we successfully induced an immunodeficient state in mini-pigs to enable the long-term accommodation of an HOBPT. The birth of OIDPs without the rejection of human cell-derived organs symbolizes an essential step toward the comprehensive evaluation of preclinical cell regeneration strategies and contributes to the development of regenerative medicine using human organs, which is expected in the near future.

## Methods

### Animals

This study used six Göttingen mini-pigs (male; age, 6–7 months; weight ≥ 15 kg; Ina MP Production Center, Oriental Yeast Co., Ltd.). The study was carried out in strict accordance with the recommendations in the Guide for the Care and Use of Laboratory Animals of the National Institutes of Health. The protocol was approved by the Institutional Animal Care and Use Committee of Nihon Bioresearch Inc. (Approval number 360484). All efforts were made to minimize suffering.

### Preparation of the HOBPT

Human normal dermal fibroblasts (HNDFBs, CC-2509) were purchased from Lonza, Inc. (Walkersville, MD, USA) and cultured in an appropriate fibroblast expansion medium with growth supplement. The cells were passaged and used within the fourth to sixth passages in this study. The cells were cultured in flasks (IWAKI T 225) and maintained at 37 °C in a humidified atmosphere containing 5% CO_2_. A cell suspension composed of HNDFB was plated into each well of an ultra-low-attachment round-shaped 96-U-well plate (Sumitomo Bakelite, Tokyo, Japan) filled with 3D culture medium. After 24 h, the cells aggregated to form a round multicellular spheroid (MCS). According to the automated measurement function equipped on our Bio-3D printer, the mean size (for several thousand MCSs) was 730.0 ± 43.3 μm ( ± standard deviation).

We used a Bio-3D Printer (Cyfuse Biomedical K.K., Japan) to assemble MCSs to construct scaffold-free tubular tissue^6^. According to a 3D structure pre-designed on a computer system, the Bio-3D Printer skewers MCSs onto needles arranged in a circle. In this system, the MCSs were aspirated from the 96-well plate by a robotically controlled fine suction nozzle and inserted into the needle array, which was made of multiple, stainless steel needles. Several thousand MCSs were crafted robotically into a 3D structure according to the pre-designed configuration. The array was removed at ~ 1 week after the placement of the MCSs onto the needle array. The configuration of the structure was retained after removal from the needle array due to fusion between the MCSs.

To encourage the tubular tissue obtained above to mature, it was mounted on a bioreactor. The bioreactor has an axle placed inside the tubular tissue, and 3D culture medium was perfused for several weeks. After perfusion, a tubular tissue of 5.0 mm in diameter and 10 mm in length was obtained (Fig. 2a).

### Transplantation of the HOBPT for a neck arteriovenous shunt

An incision was made on the left side of the neck to expose the left external jugular vein and the left common carotid artery under inhalation anesthesia of a mixed gas of N_2_O:O_2_ = 1:1 and 0.5–1.5% isoflurane. Heparin (100 IU/kg) was administered to block the left common carotid artery. End-to-end anastomosis (7–0 polypropylene continuous suture) of the HOBPT with the left common carotid artery was carried out, followed by end-to-end anastomosis to the left external jugular vein and unblocking (Supplementary Movie 1). To prevent heart failure owing to a high shunt flow rate, the left common carotid artery was ligated close to the center from the anastomotic site. The graft blood flow was measured using a flow meter, with the surgical incision closed. As antithrombotic treatment, one clopidogrel sulfate tablet (75 mg/body) and two aspirin tablets (200 mg/body) were orally administered 5 days prior to transplantation.

### Histological and immunohistochemical examinations

The tissue samples were fixed with 10% neutral buffered formalin, routinely processed, and embedded in paraffin. We performed a morphological analysis of the graft and the adjacent blood vessels on hematoxylin and eosin-stained sections by light microscopy. The collagenous matrix and elastic fibers were evaluated by Masson–trichrome staining and Elastica van Gieson staining, respectively. To analyze the migration of endothelial cells and smooth muscle cells, we performed immunohistochemical staining of endothelial markers (ERG (Biologo, ERG002-G, cloneEP111, rabbit monoclonal, undiluted) and smooth muscle markers alpha-SMA (DAKO, M0851, clone1A4, mouse monoclonal, 1:200 dilution), desmin (DAKO, IS606, cloneD33, mouse monoclonal, undiluted). Deparaffinized sections were immunostained by the avidin-biotin complex immunoperoxidase (ABC) method. To examine the fine structures of the endothelial cells, we performed transmission electron microscopy by the standard method using materials fixed with 2.5% glutaraldehyde. We counted the number of positively reactive cells against the lymphocyte markers at three hot spots (0.1 mm^2^) in the mid-portion of the HOBPT.

### Inflammatory cell and serum immunoglobulin concentrations

The numbers of neutrophil, lymphocyte, and monocyte were counted in the peripheral blood obtained from the CISP and OIDP groups after transplantation. IgM and IgG were measured by commercially available pig-specific immunoglobulin ELISA kit according to Life Diagnostics, Inc (West Chester, Pennsylvania, USA).

### Statistical analyses

Statistical analyses were performed using the JMP 11 software program (SAS Institute Inc., Cary, NC, USA). The data are expressed as the mean ± s.d. Comparisons between two groups were made using the unpaired Student’s *t* test. *P* values of < 0.05 were considered to indicate statistical significance.

### Reporting summary

Further information on research design is available in the Nature Research Reporting Summary linked to this article.

## Supplementary information

Supplementary Information

Description of Additional Supplementary Files

Supplementary Movie 1

Supplementary Data 1

Supplementary Data 2

Supplementary Data 3

Supplementary Data 4

Supplementary Data 5

Supplementary Data 6

Reporting Summary

## Data Availability

All relevant data supporting the findings of this study are either included within the article and its Supplementary Information files or are available upon request from the corresponding author.
