# Publisher Correction: Development of an immunodeficient pig model allowing long-term accommodation of artificial human vascular tubes

**DOI:** 10.1038/s41467-019-11555-5

**Published:** 2019-08-06

**Authors:** Manabu Itoh, Yosuke Mukae, Takahiro Kitsuka, Kenichi Arai, Anna Nakamura, Kazuyoshi Uchihashi, Shuji Toda, Kumika Matsubayashi, Jun-ichi Oyama, Koichi Node, Daisuke Kami, Satoshi Gojo, Shigeki Morita, Takahiro Nishida, Koichi Nakayama, Eiji Kobayashi

**Affiliations:** 1 Department of Thoracic and Cardiovascular Surgery, Faculty of Medicine, Saga University, Saga, Japan; 2 Department of Regenerative Medicine and Biomedical Engineering, Faculty of Medicine, Saga University, Saga, Japan; 3 Department of Surgical Pathology, National Hospital Organization Saga Hospital, Saga, Japan; 4 Department of Pathology & Microbiology, Faculty of Medicine, Saga University, Saga, Japan; 5 Cyfuse Biomedical K. K., Tokyo, Japan; 6 Department of Cardiovascular Medicine, Faculty of Medicine, Saga University, Saga, Japan; 7 Department of Regenerative Medicine, Kyoto Prefectural University of Medicine, Kyoto, Japan; 8 Department of Cardiovascular Surgery, National Hospital Organization Kyushu Medical Center, Fukuoka, Japan; 9 Department of Organ Fabrication, Keio University School of Medicine, Tokyo, Japan

**Keywords:** Tissue engineering, Transplant immunology, Cardiovascular biology

Correction to: *Nature Communications* 10.1038/s41467-019-10107-1, published online 21 May 2019.

The original version of this Article contained errors in Figs 2 and 3. In Fig. 2b, the scale bar label ‘500 µm’ was originally incorrectly given as ‘200 µm’. In Fig. 2d, the scale bar label ‘500 µm’ was originally incorrectly given as ‘100 µm’. In Fig. 3c, the scale bar label ‘200 µm’ was originally incorrectly given as ‘100 µm’. This has been corrected in the PDF and HTML versions of the Article.

