# Supplementary material for: Development of an immunodeficient pig model allowing long-term accommodation of artificial human vascular tubes
Source: Nat Commun. 2019 May 21;10:2244. doi: 10.1038/s41467-019-10107-1 (PMC6529409; doi:10.1038/s41467-019-10107-1)
Supplement: Supplementary file 1 — Supplementary Information [file 41467_2019_10107_MOESM1_ESM.pdf]

Supplementary Information

Development of an immunodeficient pig model allowing long-term accommodation of artificial human vascular tubes

Manabu Itoh *et al.*

Supplementary Figure 1

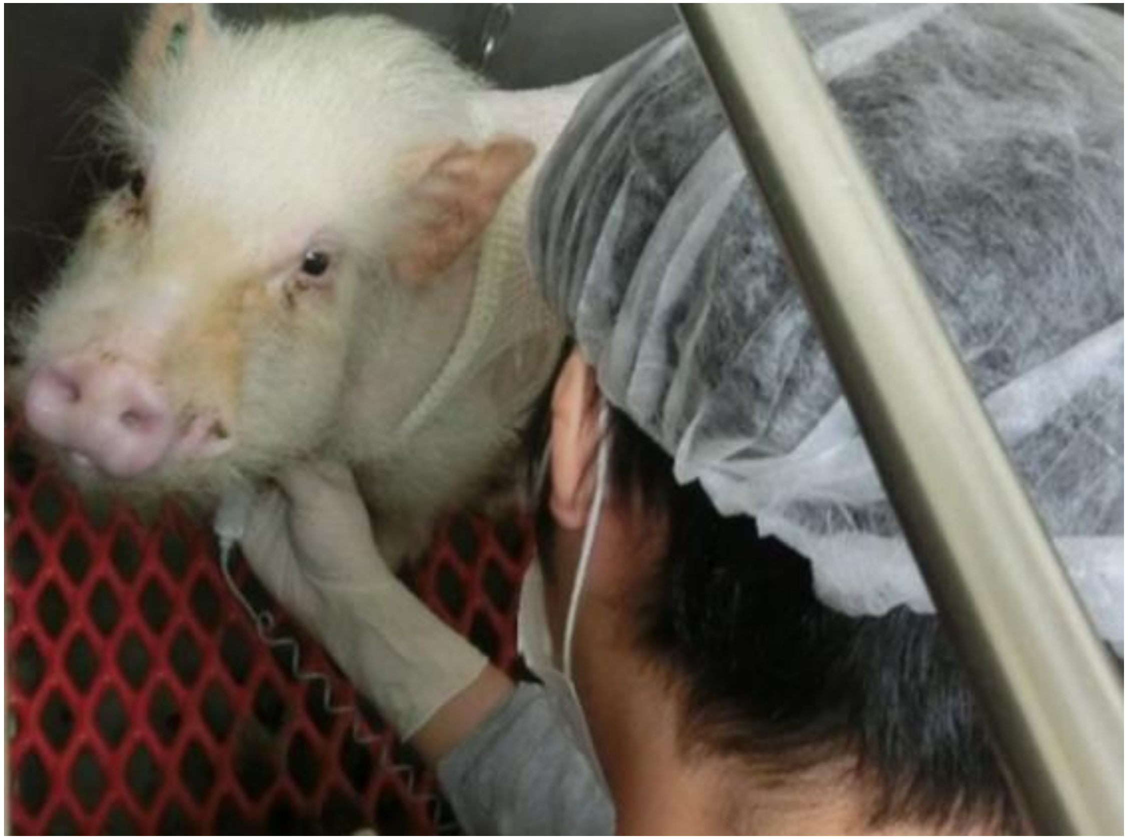

Supplementary Figure 1. Image of a mini-pig taken three months after HOBPT transplantation. The investigator was evaluating the blood flow of the HOBPT implanted as a substitute blood vessel for a neck arteriovenous shunt by ultra-sonography. HOBPT: human original 3D bioprinted tube.

Supplementary Figure 2

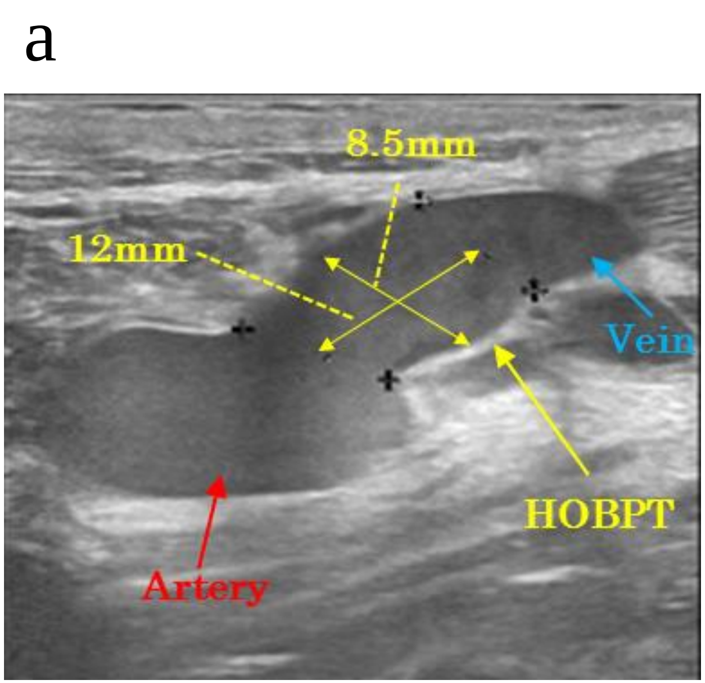

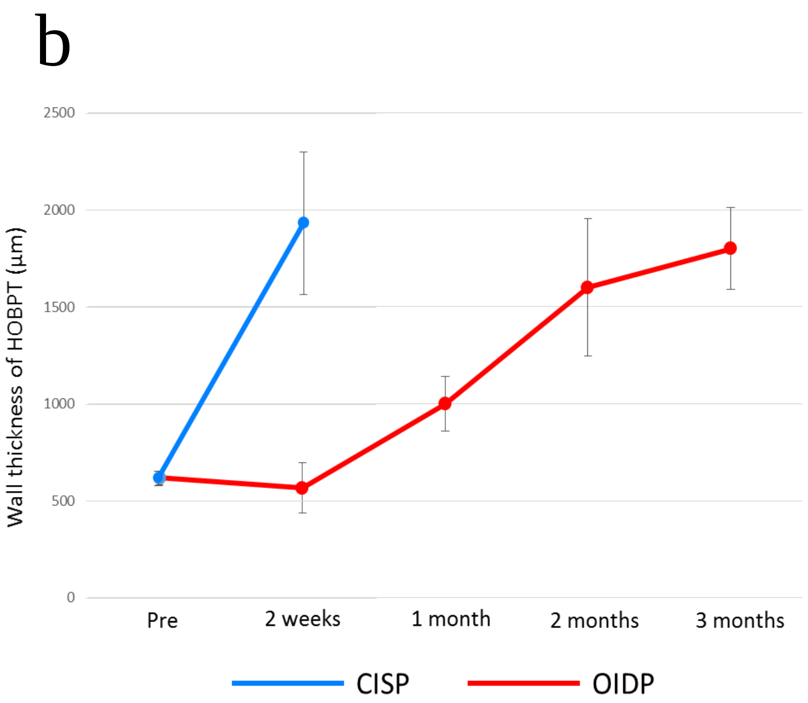

Supplementary Figure 2. An ultrasonic based morphological analysis of time-related HOBPT alterations.
In the OIDP group, an ultrasound examination revealed that the graft inner diameter was dilated from 5.0 to 8.5 mm and extended in a longitudinal direction from 10 to 12 mm at 1 month after transplantation (a).
The wall thickness of the HOBPT increased from one to three months after transplantation.
In contrast, in the CISP group, the wall thickness of the graft was increased at two weeks after transplantation (b).
(CISP group: n=6, each OIDP group: n=2). The s.d. is reported as the error bar.
HOBPT: human original 3D bioprinted tube,
OIDP: operational immunodeficient pig, CISP: conventional immunosuppressive pig.

Supplementary Figure 3

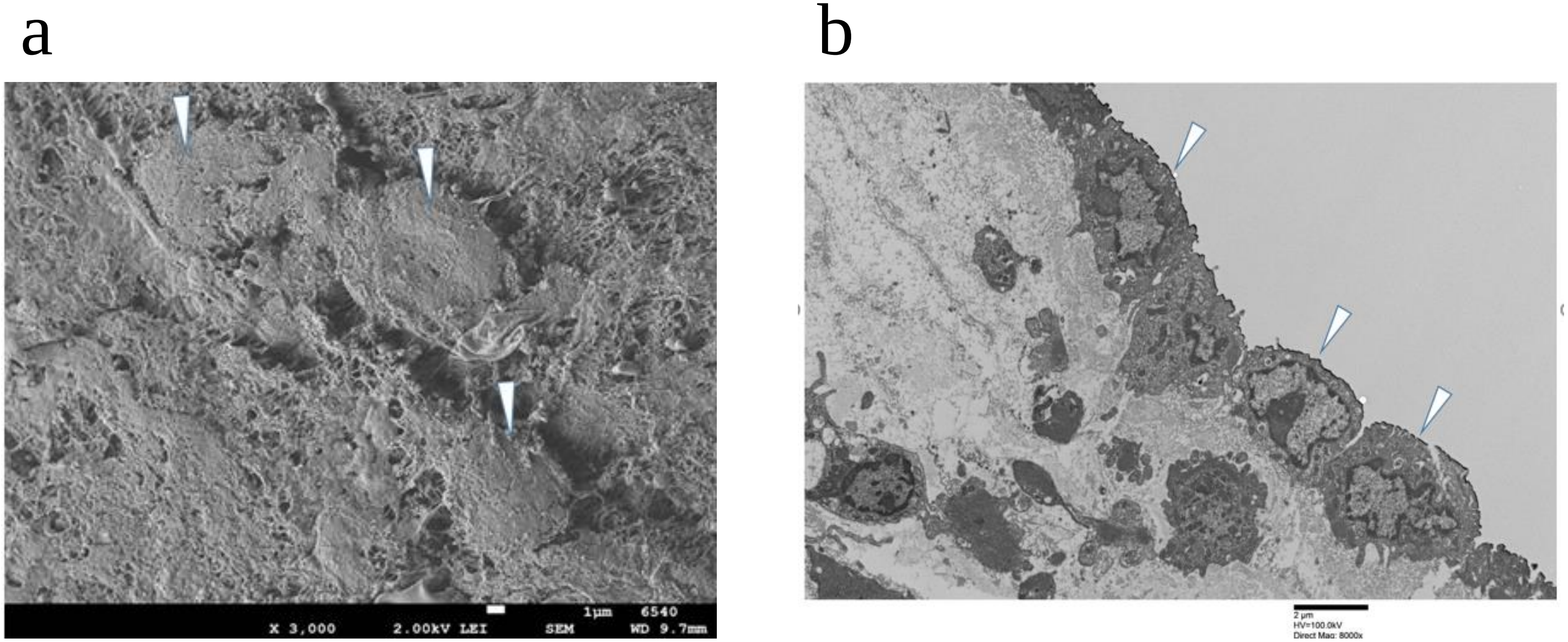

Supplementary Figure 3. Scanning and transmission electron micrographs of the luminal surface of the HOBPT at three months after transplantation.
Both photos show endothelial flat cells lining the luminal surface of the HOBPT (a, b).
HOBPT: human original 3D bioprinted tube.

Supplementary Figure 4

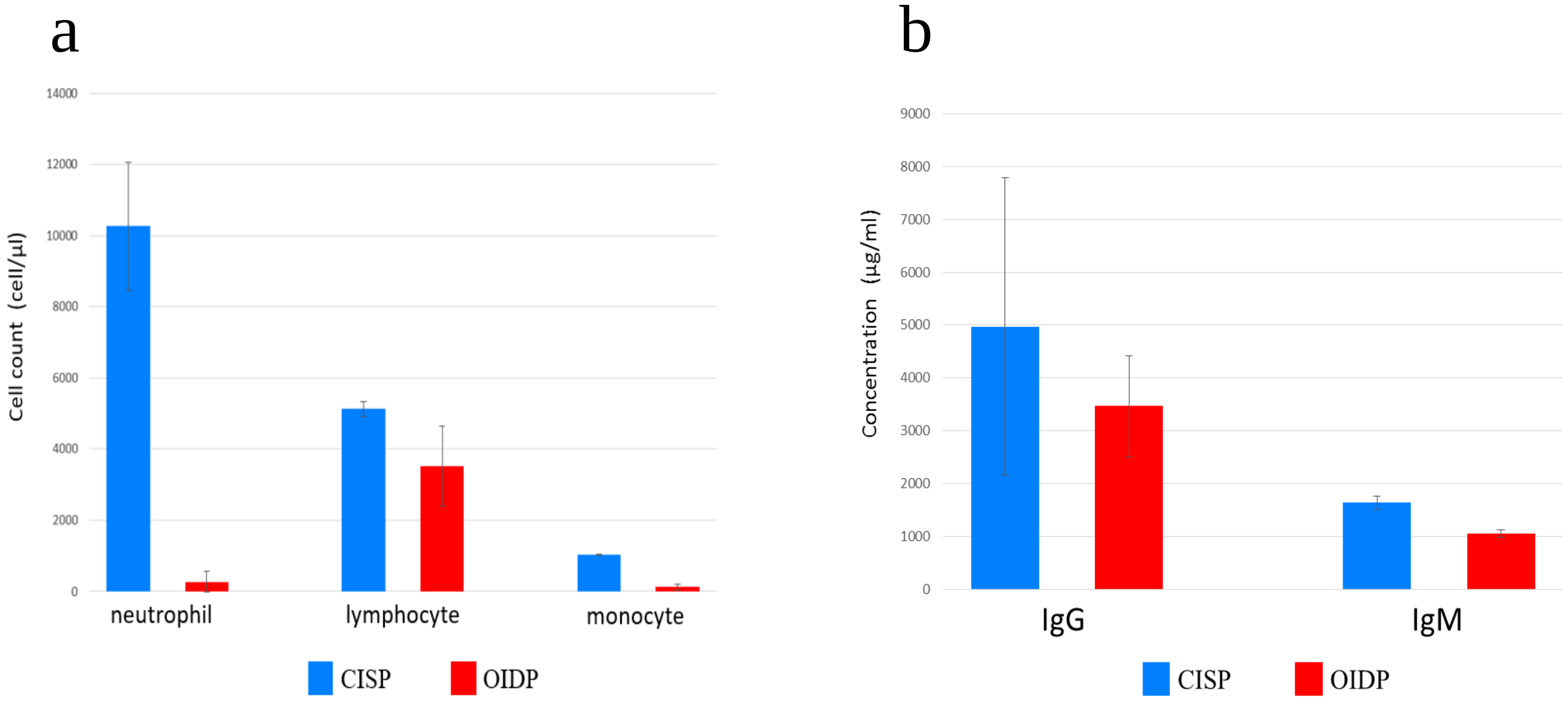

Supplementary Figure 4. Characterization of peripheral blood leukocytes and serum immunoglobulin.
Neutrophil, lymphocyte and monocyte counts in the CISP and OIDP groups at 2 weeks after implantation. (each group n= 2) (a).
Serum IgG and IgM concentrations in the CISP and OIDP groups at 2 weeks after implantation. (each group n= 2) (b).
The s.d. is reported as the error bar. OIDP: operational immunodeficient pig, CISP: conventional immunosuppressive pig.
