# Supplementary material for: Development of an immunodeficient pig model allowing long-term accommodation of artificial human vascular tubes
Source: Nat Commun. 2019 May 21;10:2244. doi: 10.1038/s41467-019-10107-1 (PMC6529409; doi:10.1038/s41467-019-10107-1)
Supplement: Supplementary file 2 — Description of Additional Supplementary Files [file 41467_2019_10107_MOESM2_ESM.docx]

**Description of Additional Supplementary Files**

**File Name:** Supplementary Movie 1.

**Description:** Transplantation of the HOBPT as a substitute blood vessel for a neck arteriovenous shunt.

**File Name:** Supplementary Data 1.

**Description:** Blood chemical findings of the OIDP model.

**File Name:** Supplementary Data 2.

**Description:** Food consumption of the OIDP model.

**File Name:** Supplementary Data 3.

**Description:** Clinical signs of the OIDP model.

**File Name:** Supplementary Data 4.

**Description:** Dosage of immunosuppressive agent of the OIDP model.

**File Name:** Supplementary Data 5.

**Description:** Blood concentration of immunosuppressive agent of the OIDP model.

**File Name:** Supplementary Data 6.

**Description:** Cytokine levels of the OIDP model.
