# Supplementary material for: Development of an immunodeficient pig model allowing long-term accommodation of artificial human vascular tubes
Source: Nat Commun. 2019 May 21;10:2244. doi: 10.1038/s41467-019-10107-1 (PMC6529409; doi:10.1038/s41467-019-10107-1)
Supplement: Supplementary file 10 — Reporting Summary [file 41467_2019_10107_MOESM10_ESM.pdf]

nature research

Corresponding author(s): Eiji Kobayashi

Last updated by author(s): Apr 15, 2019

Reporting Summary

Nature Research wishes to improve the reproducibility of the work that we publish. This form provides structure for consistency and transparency in reporting. For further information on Nature Research policies, see Authors & Referees and the Editorial Policy Checklist.

Statistics

For all statistical analyses, confirm that the following items are present in the figure legend, table legend, main text, or Methods section.

n/a	Confirmed	
☐	☒	The exact sample size (n) for each experimental group/condition, given as a discrete number and unit of measurement
☐	☒	A statement on whether measurements were taken from distinct samples or whether the same sample was measured repeatedly
☐	☒	The statistical test(s) used AND whether they are one- or two-sided<br>*Only common tests should be described solely by name; describe more complex techniques in the Methods section.*
☐	☒	A description of all covariates tested
☐	☒	A description of any assumptions or corrections, such as tests of normality and adjustment for multiple comparisons
☐	☒	A full description of the statistical parameters including central tendency (e.g. means) or other basic estimates (e.g. regression coefficient) AND variation (e.g. standard deviation) or associated estimates of uncertainty (e.g. confidence intervals)
☐	☒	For null hypothesis testing, the test statistic (e.g. F, t, r) with confidence intervals, effect sizes, degrees of freedom and P value noted<br>*Give P values as exact values whenever suitable.*
☐	☒	For Bayesian analysis, information on the choice of priors and Markov chain Monte Carlo settings
☐	☒	For hierarchical and complex designs, identification of the appropriate level for tests and full reporting of outcomes
☐	☒	Estimates of effect sizes (e.g. Cohen's d, Pearson's r), indicating how they were calculated

Our web collection on statistics for biologists contains articles on many of the points above.

Software and code

Policy information about availability of computer code

Data collection	none
Data analysis	none

For manuscripts utilizing custom algorithms or software that are central to the research but not yet described in published literature, software must be made available to editors/reviewers. We strongly encourage code deposition in a community repository (e.g. GitHub). See the Nature Research guidelines for submitting code & software for further information.

Data

Policy information about availability of data

All manuscripts must include a data availability statement. This statement should provide the following information, where applicable:

- Accession codes, unique identifiers, or web links for publicly available datasets
- A list of figures that have associated raw data
- A description of any restrictions on data availability

All relevant data supporting the findings of this study are either included within the article and its Supplementary Information files or are available upon request from the corresponding author.

Field-specific reporting

Please select the one below that is the best fit for your research. If you are not sure, read the appropriate sections before making your selection.

☒ Life sciences ☐ Behavioural & social sciences ☐ Ecological, evolutionary & environmental sciences

Life sciences study design

All studies must disclose on these points even when the disclosure is negative.

Sample size	We performed the xenotransplantation of a scaffold-free human original three-dimensional bioprinted tube onstructed entirely from human cells to the operational immunodeficient pigs (N=6) and conventional immunosuppressive pigs (N=6).
Data exclusions	none
Replication	none
Randomization	none
Blinding	none

Reporting for specific materials, systems and methods

We require information from authors about some types of materials, experimental systems and methods used in many studies. Here, indicate whether each material, system or method listed is relevant to your study. If you are not sure if a list item applies to your research, read the appropriate section before selecting a response.

Materials & experimental systems

n/a	Involved in the study	
☐	☒	Antibodies
☐	☒	Eukaryotic cell lines
☒	☐	Palaeontology
☐	☒	Animals and other organisms
☒	☐	Human research participants
☒	☐	Clinical data

Methods

n/a	Involved in the study	
☒	☐	ChIP-seq
☒	☐	Flow cytometry
☒	☐	MRI-based neuroimaging

Antibodies

Antibodies used	ERG [Biologo, ERG002-G, cloneEP111, rabbit monoclonal, predilated]), alpha-SMA [DAKO, M0851, clone1A4, mouse monoclonal, 1:200 dilution], desmin [DAKO, IS606, cloneD33, mouse monoclonal, predilated]
Validation	All of the antibodies we used are validated to react to the specific cells according to manufacturer's database. We confirmed their reactivities and specificities for each targeted cells in our pig model by positive control samples from the same model.

Eukaryotic cell lines

Policy information about cell lines

Cell line source(s)	Human normal dermal fibroblasts (HNDFBs, CC-2509), Lonza, Inc. (Walkersville, MD, USA)
Authentication	The cell line is authenticated by manufacturer's database.
Mycoplasma contamination	The cell line we used was confirmed to be negative for micoplasma contamination.
Commonly misidentified lines (See ICLAC register)	none

Animals and other organisms

Policy information about studies involving animals; ARRIVE guidelines recommended for reporting animal research

Laboratory animals	This study used six Göttingen minipigs (male; age, 6–7 months; weight ≥15 kg; Ina MP Production Center, Oriental Yeast Co., Ltd.).
Wild animals	none
Field-collected samples	none
Ethics oversight	This study was carried out in strict accordance with the recommendations in the Guide for the Care and Use of Laboratory

Ethics oversight	Animals of the National Institutes of Health. The protocol was approved by the Institutional Animal Care and Use Committee of Nihon Bioresearch Inc. (Approval number 360484).

Note that full information on the approval of the study protocol must also be provided in the manuscript.
